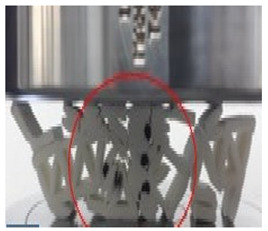# Compression Behaviour of Bio-Inspired Honeycomb Reinforced Starfish Shape Structures Using 3D Printing Technology

**DOI:** 10.3390/polym13244388

**Published:** 2021-12-14

**Authors:** S. A. S. A. Saufi, M. Y. M. Zuhri, M. Lalegani Dezaki, S. M. Sapuan, R. A. Ilyas, A. As’arry, M. K. A. Ariffin, M. Bodaghi

**Affiliations:** 1 Advanced Engineering Materials and Composites Research Centre, Department of Mechanical and Manufacturing Engineering, Faculty of Engineering, Universiti Putra Malaysia, Serdang 43400, Malaysia; syedsaufi@gmail.com (S.A.S.A.S.); sapuan@upm.edu.my (S.M.S.); 2 Laboratory of Biocomposite Technology, Institute of Tropical Forestry and Forest Product (INTROP), Universiti Putra Malaysia, Serdang 43400, Malaysia; 3 Department of Mechanical and Manufacturing Engineering, Faculty of Engineering, Universiti Putra Malaysia, Serdang 43400, Malaysia; lalegani.mr@gmail.com (M.L.D.); zizan@upm.edu.my (A.A.); khairol@upm.edu.my (M.K.A.A.); 4 Department of Engineering, School of Science and Technology, Nottingham Trent University, Nottingham NG11 8NS, UK; mahdi.bodaghi@ntu.ac.uk; 5 School of Chemical and Energy Engineering, Faculty of Engineering, Universiti Teknologi Malaysia, UTM, Johor Bahru 81310, Malaysia; ahmadilyas@utm.my; 6 Centre for Advanced Composite Materials (CACM), Universiti Teknologi Malaysia, UTM, Johor Bahru 81310, Malaysia

**Keywords:** 3D printing, bio-inspired structure, energy absorption, fused deposition modelling, honeycomb structure

## Abstract

The bio-inspired structure (e.g., honeycomb) has been studied for its ability to absorb energy and its high strength. The cell size and wall thickness are the main elements that alter the structural ability to withstand load and pressure. Moreover, adding a secondary structure can increase the compressive strength and energy absorption (EA) capability. In this study, the bio-inspired structures are fabricated by fused deposition modelling (FDM) technology using polylactic acid (PLA) material. Samples are printed in the shape of a honeycomb structure, and a starfish shape is used as its reinforcement. Hence, this study focuses on the compression strength and EA of different cell sizes of 20 and 30 mm with different wall thicknesses ranging from 1.5 to 2.5 mm. Subsequently, the deformation and failure of the structures are determined under the compression loading. It is found that the smaller cell size with smaller wall thickness offered a crush efficiency of 69% as compared to their larger cell size with thicker wall thickness counterparts. It is observed that for a 20 mm cell size, the EA and maximum peak load increase, respectively, when the wall thickness increases. It can be concluded that the compression strength and EA capability increase gradually as the cell size and wall thickness increase.

## 1. Introduction

A sandwich structure is a combination of the core structure and joint parts with layers of face sheets [1]. The honeycomb structure is one of the most common bio-inspired structures that has been studied and optimised. Different types of honeycomb structures are differentiated by their geometry shape, such as square, hexagonal, tetrahedral, pyramidal and pentagonal [2,3]. Sandwich structures have been widely used in various fields, such as aerospace and construction, due to their high strength and stiffness [4,5]. Moreover, sandwich structures can be used in 4D printing due to their specific features, such as absorbing energy [6]. These structures are well known for their excellent ability to absorb energy impacts. The hexagonal shape structure can provide superior energy absorption (EA) compared to other forms of sandwich structures under compression loading [7].

The EA of honeycomb structures can be altered to increase their crashworthiness capability. These studies showed the bio-inspired structure can increase EA [8,9]. Wang et al. [10] stated that the performance of the honeycomb structure could be enhanced by introducing a secondary structure and increasing the value of the structure’s stiffness. Another finding from Shan et al. [11] suggested that the wall thickness and cell size of the core structure could influence the failure behaviour of the sandwich structure. The multilayer hexagonal shape of the honeycomb can absorb about 31% to 60% of energy compared to the rectangular form [12]. A comparison study proved that the hexagonal shape honeycomb performed better stiffness than a foam sandwich due to the buckling and their cell size. Hence, controlling the crashworthiness of the honeycomb structure is more accessible [13]. Variation of cell size, material and wall thickness have different results on EA. By optimising these parameters, researchers can increase the EA based on the experiment conducted by Tao et al. [14]. They suggested that by changing the wall thickness, the structure had a different result on their crushing strength and EA. Aside from that, the plastic deformation near the cell wall could improve the EA by increasing the material at the intersecting area [15].

In addition, the EA capability of the cellular structure depends on the unit cell geometry, relative density, the properties of base material and the loading force [16,17]. Xu et al. [18] studied the EA capability between the standard hexagonal shape with a combination of hybrid structures consisting of auxetic and hexagonal honeycomb cells under in-plane compression. The hybrid structure improved by 38% compared to the standard hexagonal honeycomb structure. Moreover, Wang et al. [19] worked on the inhomogeneity honeycomb structure. One of the factors that could influence the structure failure during the compression is the regularity and adhesive failure of cell size. Almost all failures of honeycomb structures during compression occur near the joint, where the cells are connected. It creates an over-stiff area with a higher stress concentration [20]. It is shown that the capability of reinforcement in honeycomb structures had a higher significant result in terms of strength compared to the typical geometry structures. Moreover, the elastic modulus and the EA enhanced by 26% and 73%, respectively [21]. Another study showed the integration of reinforced structure not only increased the EA but also increased the peak load. Sun et al. [22] investigated a combination of sandwich structures reinforced with a grid at higher peak loads.

According to Tuo et al. [23], a honeycomb structure integrated with a plate made of basalt fibre, compressed under an edgewise position, proved that the structure had a larger shear modulus of elasticity and good plastic deformation ability. Lei et al. [24] examined the edgewise compression to investigate the behaviour of reinforced fibreglass honeycombs columns. The results showed that the load decreased with an increase of the columns’ length. Similarly, Kara et al. [25] stated that less deflection occurs when a higher load is exerted, and they suggested reinforcing the honeycomb structure for major improvement. Another study on the comparison of edgewise and flatwise testing positions found that the former position for sandwich beam exhibited higher stiffness and strength bearing capacity compared to the latter position [26]. There are three phases that a structure undergoes during the compression, which are linear region, plateau region and densification. The phases are defined through a compression test in a stress-strain curve or load-displacement curve. The structure starts to fail when a continuous load presses the sample over the elastic region.

Meanwhile, recent developments and rapid usage of additive manufacturing (AM), mostly on fused deposition modelling (FDM) technology, has led to more possibilities to fabricate and design complex products [27,28]. Three-dimensional printing technology is capable of producing a wide range of structures, from simple to complex shapes [29]. The nature of the FDM process is to fabricate products layer by layer [30,31]. A barrage of materials from polymer to composite can be used in this process, which is in the shape of filament [32]. Polylactic acid (PLA) is a friendly biodegradable material that is made of starch and is converted into dextrose, a fermentable sugar by enzymatic hydrolysis, a process where the micro-organisms break the starch into smaller pieces called lactic acid [33,34]. In a study performed by Rebelo et al. [35], the EA of a sacrificial honeycomb cladding was analysed by using PLA material. The findings concluded that the dynamic force and specific energy absorption (SEA) were directly proportional to the relative density, which controls the buckling of the interior cell wall and the deformity of the structure for the top and bottom layer.

Chen et al. [36] studied a hierarchical honeycomb under large compressive deformation. Due to the complexity of their specimens, the products were made by a 3D printer and were tested under compression loading. To validate the experiment result, samples were analysed in FEA, and the results were similar. Meanwhile, Kucewic et al. [37] worked on the deformation behaviour, and failure of 3D printed cellular structures and compared their experimental findings with the numerical analysis. Moreover, Sahu et al. [38] studied the effect of the cell size of high-density polyethylene (HDPE) sandwich honeycomb structures made with the 3D printing process. The results indicated that a smaller cell size inhabited a larger SEA. As the cell size increased, the crushing force efficiency (CFE) value is decreased, while opposite results were found with a constant cell size with a different wall thickness. In addition, a study on hierarchical honeycomb under a larger compressive deformation is conducted, where the honeycomb wall is replaced by a triangular lattice. The honeycomb showed an improvement in stiffness and EA [36].

This study aims to improve the compression properties of the current honeycomb structure with reinforcement of a bio-inspired starfish shape. Aside from that, the main objectives of this study are to investigate the energy capability of bio-inspired structures, such as their EA, SEA and CFE, fabricated by using FDM technology, as well as their failure deformation under quasi-static loading conditions, and it focuses on the effect of cell size and cell wall thickness.

## 2. Material and Methods

### 2.1. Material

The material used is polylactic acid (PLA) supplied by PolyLiteTM Polymaker, (Shanghai, China) with the same properties: density ranging from 1.17 to 1.24 g/cm^3^ and the melting temperature between 190 and 230 °C. The materials come in white and black in colour.

### 2.2. Methods

**Figure 1:**
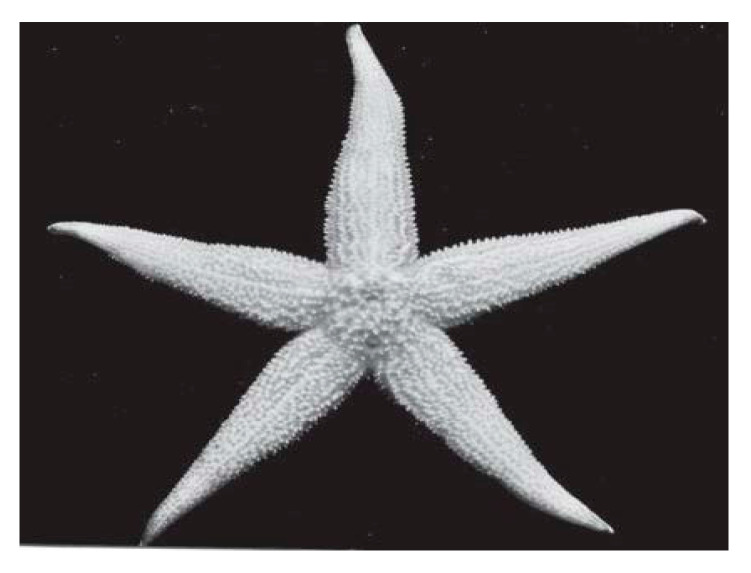
Starfish shape [41].

**Figure 2:**
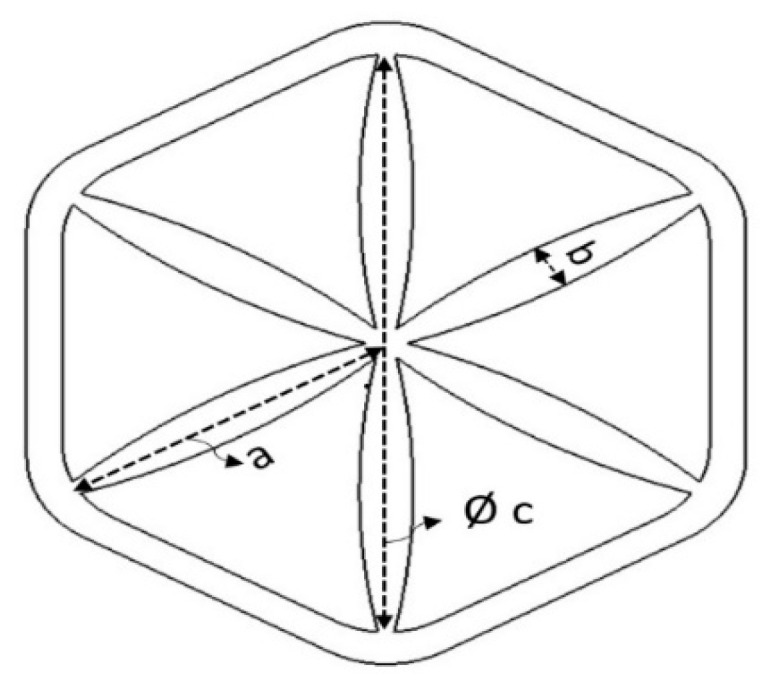
Illustration of honeycomb reinforced starfish structure.

The bio-inspired structures are based on the combination of honeycomb and starfish structures (see Figure 1). The internal structure of the honeycomb is reinforced with the inspired structure of starfish. It is modified by adding 6 branches of the starfish for better stability and symmetry. The design of the combined structure is drawn using Solidworks^®^ software (Version 2010) before converting into the standard tessellation language (STL) format for the 3D printing process. Figure 2 illustrates the complete honeycomb reinforced starfish structure. The curve design of the branch is used to reduce the load at the centre and distribute the load to the edge, as suggested by Wang et al. [39]. Each branch has the same angle with the value of 60°, (a) is the radius of the starfish shape, (b) thickness of the starfish shape (which is equivalent to the honeycomb wall thickness) and (c) is the honeycomb cell size. Prior to conducting testing under quasi-static loading, three sets of samples for each size and thickness are prepared, which is similar to Khan [40].

**Figure 3:**
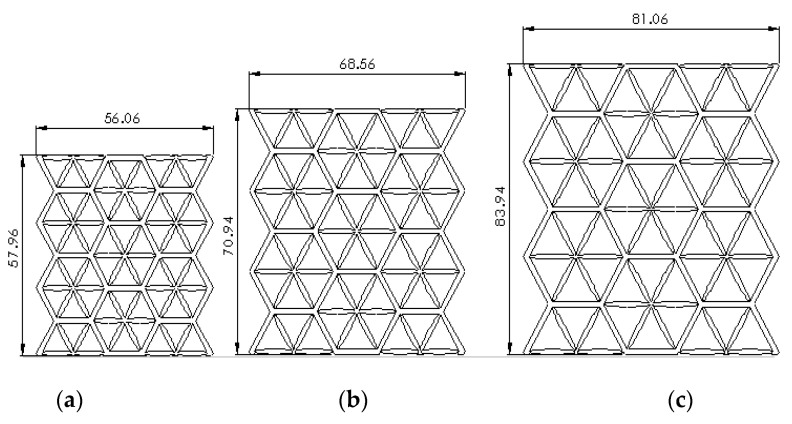
Cell size of (**a**) 20, (**b**) 25 and (**c**) 30 mm.

**Figure 4:**
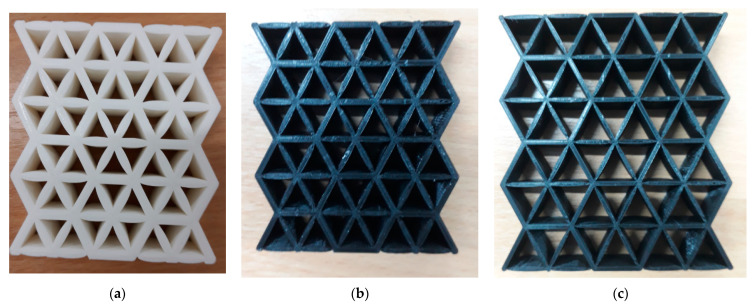
The final 3D printed specimens for (**a**) cell size 20 mm, thickness 2.5 mm, (**b**) cell size 25 mm, thickness 2 mm and (**c**) cell size 30 mm, thickness 1.5 mm.

Figure 3 shows the samples with different cell sizes, where all have the same width of 15 mm. Here, the controlled parameter is the cell size, these being 20, 25 and 30 mm. Each size has 3 different wall thicknesses, which are 1.5, 2.0 and 2.5 mm. Table 1 presents the physical properties of the 3D printed structures. The model of the FDM machine used is Ultimaker 2+ (Utrecht, The Netherlands). The nozzle diameter is 0.25 mm, and the nozzle temperatures are between 190 and 230 °C, respectively. The layer thickness is 60 microns with a built-up speed of 60 mm^3^/s. The build orientation is set to 0° due to the optimum angle in the printing process. The final 3D printed samples are shown in Figure 4.

The ASTM standard D7336/D7336M recommends three criteria of energy absorption. The capability of the structure to resist deformation from an external force depends on its energy absorption. The energy absorption capability of the cellular structure depends on the unit cell geometry, relative density, the properties of base material and the loading force [17]. Energy absorption can be obtained by determining the area under the load-displacement curve. It is derived from the mathematical formula as follows:

(1) $$\mathrm{EA}=\overset{d}{\underset{0}{\int }}F\left(x\right)\mathrm{dx}$$

where the F(x) is the function of displacement x and d is the deformation. Total energy absorption is the accumulative load-displacement curve from zero to the maximum deformation. Specific energy absorption (SEA) is derived from the value of energy absorption per mass.

(2) $$\mathrm{SEA}=\frac{\mathrm{EA}}{m}$$

Crushing force efficiency (CFE) analysis is the performance of the structure during the compression process. It is the ratio of the average load in the plateau region to the load at the maximum peak load. As explained from the definition in ASTM D7336, the mathematical expression of CFE is obtained as follows:

(3) $$\mathrm{CFE}=\frac{F_{\mathrm{ave}}}{F_{\max}}$$

where F_ave_ is the average load in the plateau region and F_max_ is the maximum peak load.

**Figure 5:**
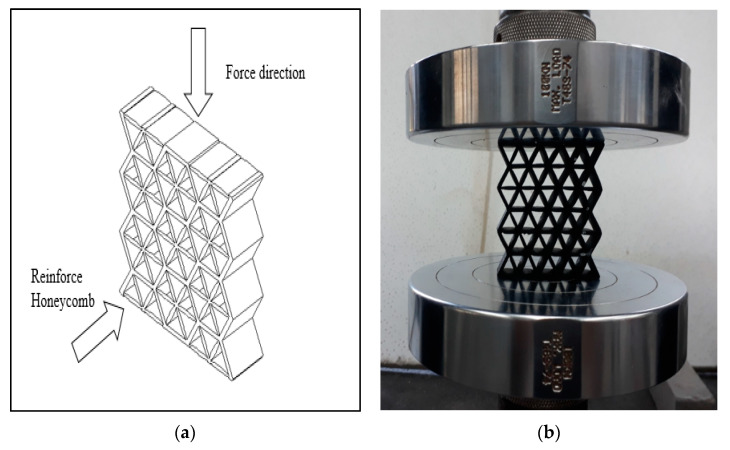
(**a**) Schematic of sample orientation during test. (**b**) Sample orientation during test.

Following this, an edgewise compression test is performed following the ASTM C364 standard. An INSTRON 3382 universal testing machine is used to conduct the compression test with a load cell of 100 kN and crosshead displacement of 1 mm/min. The orientation of the sample is facing perpendicular to the force exerted, as shown in Figure 5. This method is similar to Ivañez et al. [16]. The four stages of the structure are photographed at every 5 mm of displacement to determine its failure deformation.

## 3. Results and Discussion

The results obtained from the compression test are plotted in the form of a load-displacement curve. The maximum peak means the maximum value of loading that the structure can resist. The value of the stress determines the strength capability of the structure. Here, the maximum peak value of the tested samples is determined from the highest value stress in the linear region. The CFE is taken from the load-displacement curve. It is calculated from the ratio between average forces in the plateau region and the maximum peak value. Meanwhile, the EA value is calculated based on the area under the load-displacement curve. The failure deformation and the reaction of the structure during the experiment are also investigated. In this study, the maximum peak value is discussed in two ways. First, the comparison under variable cell sizes with constant wall thickness and secondly constant cell size with variable wall thicknesses. This work focuses on investigating the effects of cell size with variable wall thicknesses and vice versa. For constant wall thickness, the sample with the highest maximum peak load is used to compare with the other results.

### 3.1. Effects of Cell Size on the Structure

**Figure 6:**
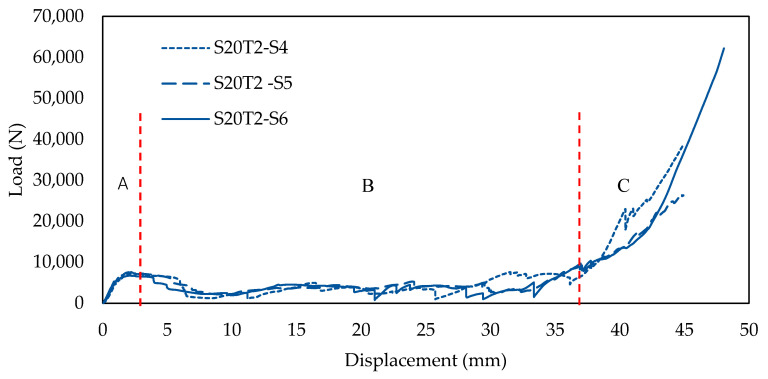
The load-displacement curve for samples with 20 mm cell size with 2 mm wall thickness.

Figure 6 presents the experimental result for a batch of samples with a 20 mm cell size and a 2 mm wall thickness. It is observed that the structure failed in three stages, these being uniform compression and elastic instability (A), plastic deformation (B) and plastic instability (C). In section A, a linear region is observed where the load increases proportionally with an increase in displacement. This is where the reinforced starfish shape structure provides some additional support to the main honeycomb core. At the end of this section (maximum load), the sample started to collapse and deformed due to the breakage of the starfish shape joint from the honeycomb core (as shown in Table 2). As the loading continued, the structure started to lose its stability and the load decreased gradually due to the less interaction of the reinforced starfish shape to its main structure. Thus, this leads to the plateau region loading under section B, which shows a constant loading compared to sections A and C, where the main structure (honeycomb) takes its role to support the overall structure with minimal support from the secondary support (starfish shape). In section C, the structure is fully collapsing, where the region is called densification, where the structure is no longer able to withstand any force.

**Figure 7:**
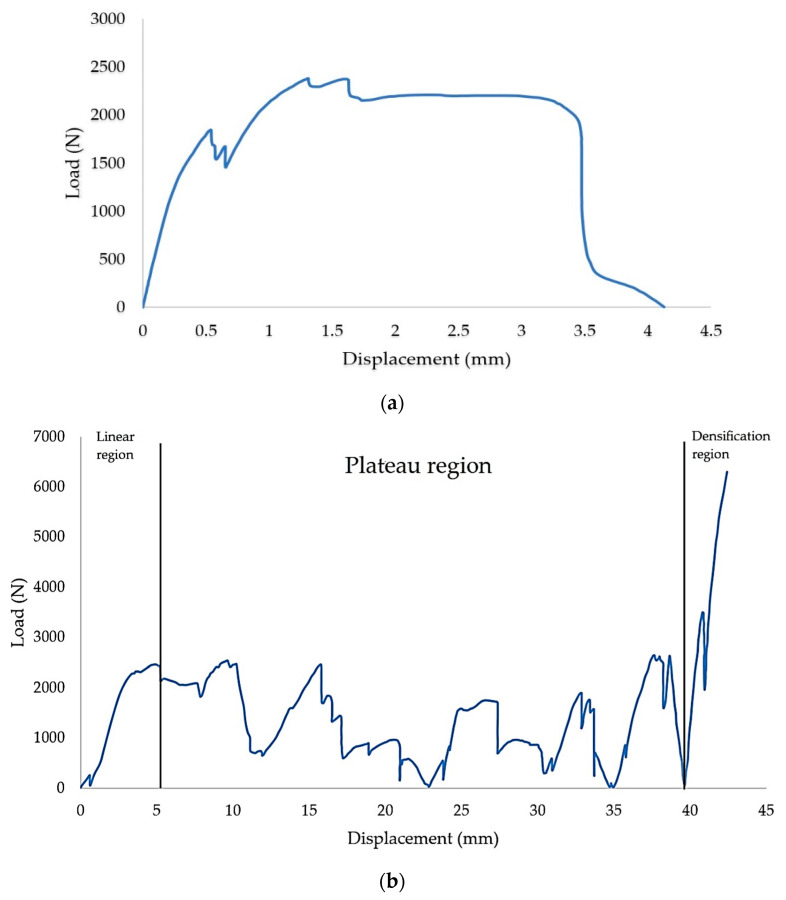
Load-displacement curve of a sample with a 25 mm cell size, 2 mm wall thickness, showing the (**a**) initial stage and (**b**) second stage of the failure.

However, the sample with a 25 mm cell size and wall thickness of 2 mm showed different behaviour compared to other samples. Here, the sample is likely to behave as two different structures. At the first stage, the loading started to decrease after a displacement of 3 mm, as shown in Figure 7a. Following this, the loading starts to increase again. This indicates that the structure has entered the second stage of the compression loading, where it starts from zero again before continuing with linear and plateau regions and finally the densification region (see Figure 7b). At the first stage, the failure is due to the breakage of the link between the structure at both sides of the structure, which causes the decrease of the compression loading. As the load continued, the bottom side of the collapsed branch between the honeycomb structure started to overlap each other and formed a ‘new’ structure. In contrast, most of the structural centre parts remain in their original form.

During the plateau region, the side structure started to break away from the honeycomb structure. Hence, the structure lost support from the edge side. As the load pressed, the side of the structure continued to move further from the main structure. Simultaneously, in the middle of the structure, the branch (see red circle in Table 2) started to break. Meanwhile, at 30 mm of displacement (Plateau region), the wall thickness at the centre structure split and became thinner. The split part started to fold at 40 mm displacement (densification) as the structure height compressed. The link that connected to the honeycomb started to break from the joining, and a major loss happened for support. Moreover, at this stage, inhomogeneity of cells occurred. This resulted in adhesive failure between the cell sizes [19]. A failure is also recorded at the middle of the core structure, where the starfish shape broke from the honeycomb structure. In the densification region, only the centre of the structure formed a solid structure.

**Figure 8:**
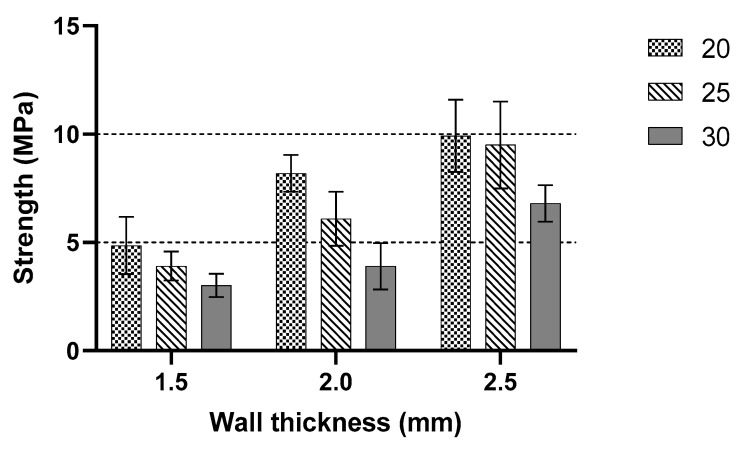
Comparison between constant wall thicknesses with variable cell sizes.

Table 2 shows a comparison of structure deformation between the cell size of 20 mm with 2 mm wall thickness and the cell size of 25 mm with 2 mm wall thickness. From the observation, the behaviour of both structures at initial and maximum peak values showed no differences. The geometry for both structures showed no sign of rupture, buckling or break. Due to the continuous load, in the collapsed structure, a solid form structure started to develop mainly on the bottom side. For the cell size 25 mm with 2 mm wall thickness, an internal tear started to develop during the densification region. Slicing the link of the structure in half and a portion of the structure broke from the main structure. At the densification area, a 20 mm cell size with 2 mm wall thickness showed a larger solid area compared to the 25 mm cell size with 2 mm wall thickness. It is observed that the structure of 20 mm cell size with 2 mm wall thickness started to reach its densification point at the displacement of 40 mm. On the other hand, the 25 mm cell size with 2 mm thickness started its densification at the displacement of 45 mm. The split that occurred in the structures is due to the kink-band failure [42]. The main reason might be due to the shortened infill during the process, as stated by Jerez-Mesa et al. [43]. They stated that the infill density could influence the fatigue lifespan of the PLA material. It is also found that the type of infill can be contributed to the strength of the printed structure [44]. The collapse sequences were almost similar for all samples except the cell size of 25 mm with 2 mm wall thickness. Increasing cell size reduced the maximum peak value [15]. Figure 8 gives information about the wall thicknesses of 1.5 to 2.5 mm. The maximum peak value decreased gradually as the cell size increased. Smaller cell size exhibited a larger peak value compared to the larger cell size, regardless of the wall thickness size.

### 3.2. Effects of Wall Thickness on the Structure

**Figure 9:**
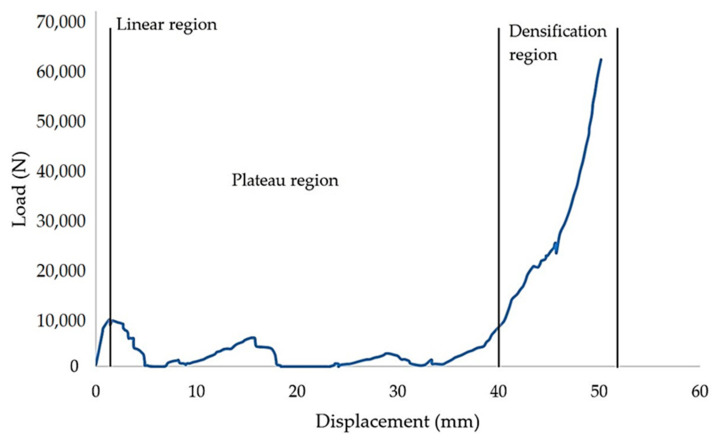
Cell size 20 mm, wall thickness 2.5 mm load-displacement mean curve.

Figure 9 illustrates a load-displacement graph for 20 mm cell size with 2.5 mm wall thickness. Table 3 shows the collapse behaviour in the plateau region. The structure started to collapse after reaching the maximum peak value. The load pressed the structure, and samples started to expand to the edge side. At 5 mm displacement, the link between the cell size at the top upper side started to fracture and detach from the main structure; however, the centre of the part still maintained its shape (see stage I). After it reached 10 mm of displacement, the edge side of the structure started to move to the outer part of the structure. It can be seen the fracture that broken section parts move further away. At the same time, the starfish structure inside the honeycomb had a failure breaking and created an almost solid shape at the top centre and the edge sides.

As the displacement reached 15 mm, there was no significant shape-changing at the bottom side, while the centre part of the structure became thick. A portion in the top right side of the structure breaks from its main structure. As shown in the graph, the load decreases at 20 mm displacement until 25 mm displacement. The load increases at 30 mm displacement. Then, the centre of the part becomes more solidified, even though some sections from the left of the sample move away from the main structure. The left side of the structure collapsed from the main structure. The structure started to become almost solid at 40 mm displacement and indicated that the structure entered the densification region. Papka and Kyriakides [45] concluded their finding by defining the elastic region. The relationship of load and displacement is quite uniform, and the behaviour of the geometry is fairly stiff. As the load reached the highest peak, a sharp fall happened in the load-displacement chart subsequently.

**Figure 10:**
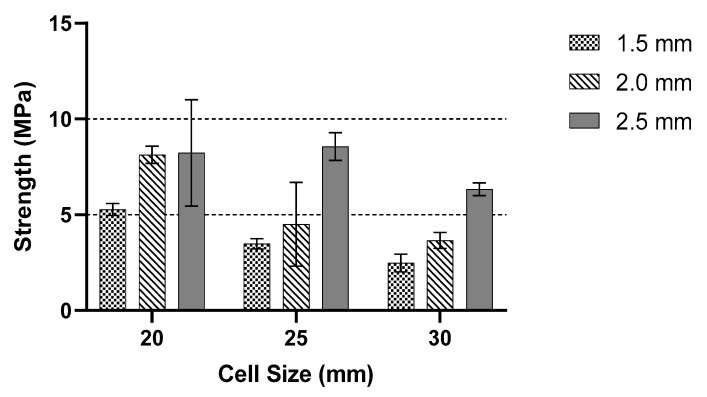
Comparison between constant cell sizes with variable thickness.

From the analysis of the maximum peak value for the three cell sizes with a different wall thickness, a comparison between 20, 25 and 30 mm cell sizes for the highest peak value is shown in Figure 10. The cell size with the largest wall thickness had the highest maximum value compared to the smaller cell size. The wall thickness of the structure affected the peak load value. This bar chart shows a comparison between samples with various cell sizes and wall thicknesses. The experimental data showed an average of 5.28 MPa stress for 1.5 mm thickness with 20 mm cell size. Moreover, all samples showed an almost identical value accordingly. As the wall thickness increased to 2 mm, the average peak value increased with an average of 8.13 MPa for 20 mm cell size. The 25 mm cell size sample showed an almost similar pattern to the 20 mm cell size. Except for 2.0 mm thickness, one of the samples showed a large different record with 3.06 MPa of peak value compared to the other two samples, 6.30 and 5.16 MPa, respectively. Overall, the maximum peak value increased as the cell size increased. The 25 mm cell size trend showed that an increase in wall thickness caused the peak value to change proportionally. For a cell size of 30 mm, the same trend is recorded as the other two cell sizes. As the wall thickness increased, the value of the maximum peak value increased gradually. However, from the obtained data, the values of 30 mm were smaller than 20 and 25 mm, which were similar to the Shan et al. [11]. The peak stress decreased with an increase in sample size. The highest stress value among samples is recorded around 6.67 MPa for the 30 mm cell size, 8.39 MPa for the 20 mm cell size and 8.79 MPa for the 25 mm cell size.

### 3.3. Effects of Bio-Inspired Structure on Energy Efficiency and Energy Absorption

**Figure 11:**
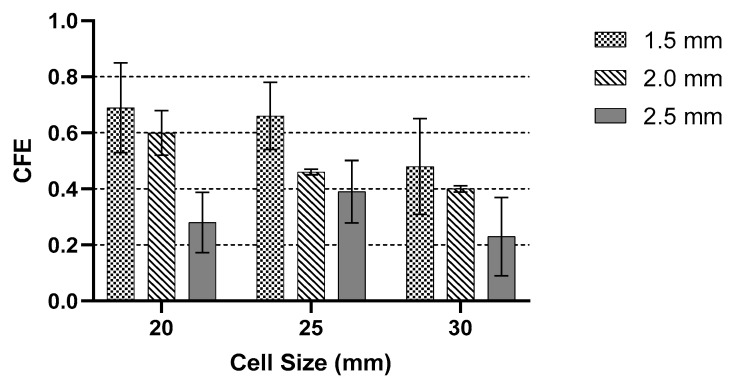
Comparison of CFE with different cell sizes and wall thickness.

Figure 11 shows the CFE values of cell sizes 20, 25 and 30 mm, respectively. Based on the experimental result, the 20 mm cell size with a lower wall thickness exhibited a higher ratio average force in the plateau region to peak force, which is near to 0.7, compared to 2.5 mm wall thickness with a ratio of 0.28. The peak load at 2.5 mm is much higher than 1.5 mm, but the average load in the plateau region in 1.5 mm is higher compared to 2.5 mm wall thickness. Different behaviour is recorded in the 25 mm cell size, where 2.5 mm wall thickness recorded a higher peak load compared to 20 mm cell size. However, the CFE values were still lower than the 1.5 mm wall thickness. The CFE value decreased as the wall thickness increased, similar to the findings by Ivañez et al. [16]. As the cell size decreased, the CFE value decreased as well. The highest CFE occurred when the cell size was 20 mm with 1.5 mm wall thickness, which was the smallest sample.

**Figure 12:**
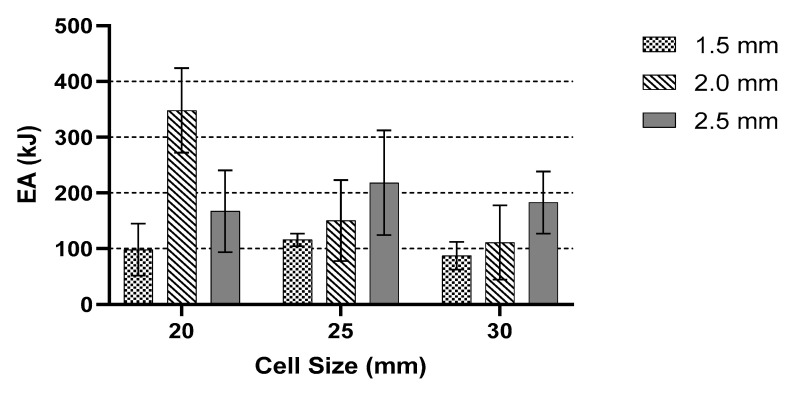
The comparison of EA for the same cell size with different wall thicknesses.

**Figure 13:**
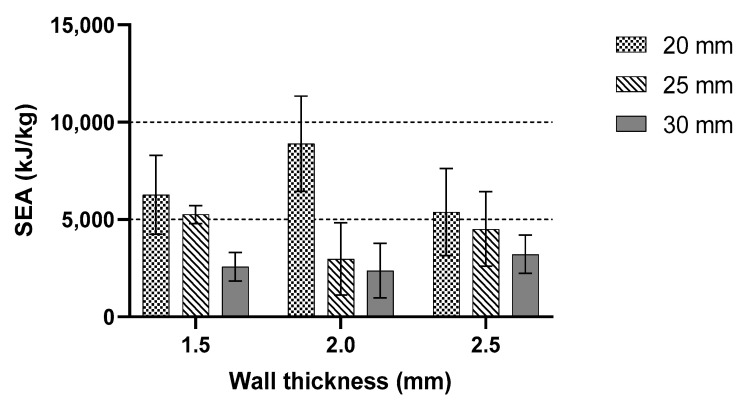
The SEA comparison of the same wall thickness with different cell sizes.

The result for the 30 mm cell size showed a similar pattern to the 20 mm and 25 mm cell sizes. Figure 12 and Figure 13 show the results for EA and SEA for all specimens, respectively. From the given result, the highest average EA and SEA occurred where the wall thickness was 2 mm with a 20 mm cell size. The highest value was recorded at 348.18 kJ of EA and 8356.21 kJ/Kg for the SEA. Furthermore, the difference between all three samples with 2.0 mm wall thickness was quite large. Overall, the given values for both EA and SEA were lower than the 20 mm cell size. Observing the highest value of both EA and SEA for the 25 mm cell size in this study showed that the wall thickness is an important factor. As an example, a major improvement with the value of 71% can be seen in the sample with 20 mm cell size and a 2.0 mm thickness compared to those with 30 mm cell size and 1.5 mm thickness.

Figure 12 summarises a comparison of EA in terms of cell size, of which the highest values of EA are selected. In brief, a larger cell size generated a smaller EA. From the graphical chart, the behaviour between 25 and 30 mm cell sizes is similar. As the wall thickness increased, the amount of EA decreased. In addition, Figure 13 shows a comparison between the SEA values in terms of wall thickness, which present a similar pattern as in Figure 11. Based on the analysis for maximum peak force, CFE, EA and SEA, the 20 mm cell size with a 2 mm wall thickness showed a remarkable result compared to the rest of the samples. Moreover, similar experimental setups and testing parameters from previous studies are used. The results from Xiang and Du [46] show the SEA and EA of different thicknesses and cell sizes of bio-inspired structures. The results, after adding the bio-reinforcement, increased by 35.97%. Thomas and Tiwari [15] studied the behaviour of aluminium honeycomb reinforced structure, where the sample sizes were 10 mm in cell size with a 0.1 mm wall thickness. The samples were strengthened by a secondary reinforcement structure. An EA produced by an aluminium reinforcement structure had a higher value than ABS honeycomb structure from the study by Kucewicz et al. [20]. It is shown that an additional secondary structure as a reinforced structure could increase the EA capability of the common honeycomb structure. Another study is conducted on a novel bio-inspired honeycomb sandwich panel (BHSP) based on a woodpecker’s beak. They found out the SEA of this novel structure is higher by 125% and 63.7% compared to the conventional sandwich panel. They recorded different values for each sample with different thicknesses. The same results happened in this study compared to the recorded experiments.

## 4. Conclusions

A range of compression testing was conducted to analyse the strength and EA capability of a starfish-reinforced honeycomb shape structure. The effect of cell size and wall thickness was investigated. To achieve a higher peak load, a larger cell size and wall thickness offered the optimum criteria. In this case, a comparison between the wall thicknesses presented a uniform pattern. As the wall thickness increases, the peak load increases, regardless of the core size. On the other hand, as the cell size increases, the peak load will decrease. The compression behaviour of the smallest wall thickness gives better performance than the larger wall thickness. As the wall thickness increases, the efficiency decreases (regardless of the cell size). In addition, the results indicated that a higher CFE value was generated from a smaller value of energy absorption. In brief, as the wall thickness increases, the CFE value will decrease, yet the EA is increased. For future works, a study on the structural joint or node can be further investigated to increase its performance, as most of the structure failed at the interlink between cell size. Their capability under dynamic loading conditions should also be considered.

## Figures and Tables

**Table 1:** Physical properties of the 3D printed samples.

Cell Size (mm)	Sample No.	Height (mm)	Length (mm)	Width (mm)	Wall Thickness (mm)	Weight (g)
20	S1	57.96	56.06	15	1.5	23
	S2	57.96	56.06			23
	S3	57.96	56.06			23
	S4	59.96	58.08		2.0	31
	S5	59.96	58.08			31
	S6	59.96	58.08			31
	S7	61.96	60.11		2.5	38
	S8	61.96	60.11			39
	S9	61.96	60.11			38
25	S10	70.94	68.56		1.5	28
	S11	70.94	68.56			29
	S12	70.94	68.56			29
	S13	72.94	70.58		2.0	39
	S14	72.94	70.58			39
	S15	72.94	70.58			39
	S16	74.94	72.61		2.5	49
	S17	74.94	72.61			48
	S18	74.94	72.61			48
30	S19	83.94	81.06		1.5	34
	S20	83.94	81.06			34
	S21	83.94	81.06			34
	S22	85.94	83.08		2.0	46
	S23	85.94	83.08			47
	S24	85.94	83.08			47
	S25	87.94	85.11		2.5	57
	S26	87.94	85.11			57
	S27	87.94	85.11			59

**Table 2:** Comparison of structure failure deformation under different cell sizes.

Stage	20 mm Cell Size 2.0 mm Wall Thickness	25 mm Cell Size 2.0 mm Wall Thickness
I (Initial stage)	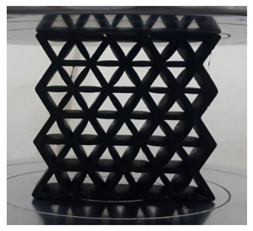	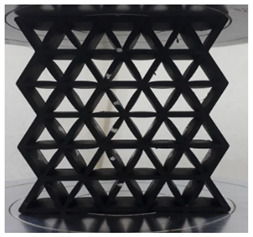
II (Max peak)	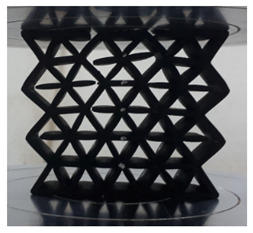	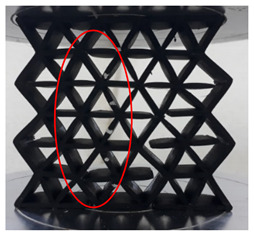
III (Plateau region)	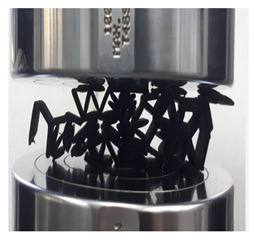	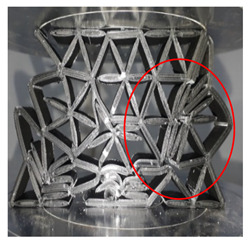
IV (Densification)	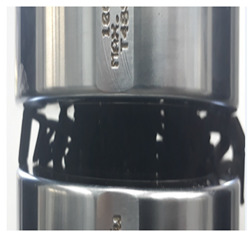	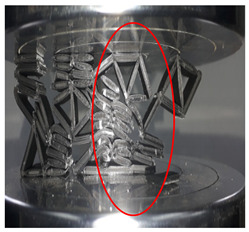

**Table 3:** Failure sequence of the structure with 20 mm cell size and 2.5 mm wall thickness.

Stage	20 mm Cell Size and 2.5 mm Wall Thickness
I (Initial stage)	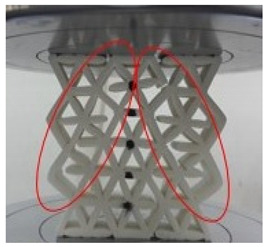
II (Max peak)	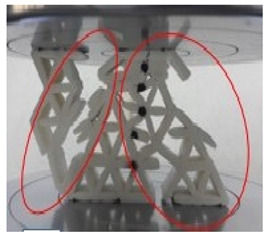
III (Plateau region)	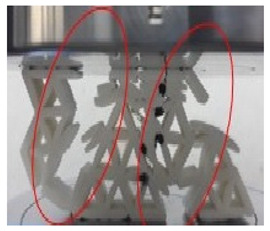
IV (Densification)